# Poly(3-hydroxybutyrate) (PHB) and Polycaprolactone (PCL) Based Blends for Tissue Engineering and Bone Medical Applications Processed by FDM 3D Printing

**DOI:** 10.3390/polym15102404

**Published:** 2023-05-22

**Authors:** Štěpán Krobot, Veronika Melčová, Přemysl Menčík, Soňa Kontárová, Michala Rampichová, Věra Hedvičáková, Ema Mojžišová, Andrej Baco, Radek Přikryl

**Affiliations:** 1 Institute of Material Chemistry, Faculty of Chemistry, Brno University of Technology, Purkyňova 464/118, 612 00 Brno, Czech Republic; 2 Institute of Experimental Medicine, CAS, Vídeňská 1083, 142 20 Praha, Czech Republic; 3 Institute of Natural and Synthetic Polymers, Faculty of Chemical and Food Technology, Slovak University of Technology in Bratislava, Radlinského 9, 812 37 Bratislava, Slovakia; 4 PANARA, a.s., Krškanská 21, 949 05 Nitra, Slovakia

**Keywords:** 3D printing, FDM, poly(3-hydroxybutyrate), bone tissue engineering, scaffold, biocompatibility, polycaprolactone, polylactic acid

## Abstract

In the presented work, poly(3-hydroxybutyrate)–PHB-based composite blends for bone medical applications and tissue engineering are prepared and characterized. PHB used for the work was in two cases commercial and, in one case, was extracted by the chloroform-free route. PHB was then blended with poly(lactic acid) (PLA) or polycaprolactone (PCL) and plasticized by oligomeric adipate ester (Syncroflex, SN). Tricalcium phosphate (TCP) particles were used as a bioactive filler. Prepared polymer blends were processed into the form of 3D printing filaments. The samples for all the tests performed were prepared by FDM 3D printing or compression molding. Differential scanning calorimetry was conducted to evaluate the thermal properties, followed by optimization of printing temperature by temperature tower test and determination of warping coefficient. Tensile test, three-point flexural test, and compression test were performed to study the mechanical properties of materials. Optical contact angle measurement was conducted to determine the surface properties of these blends and their influence on cell adhesion. Cytotoxicity measurement of prepared blends was conducted to find out whether the prepared materials were non-cytotoxic. The best temperatures for 3D printing were 195/190, 195/175, and 195/165 °C for PHB-soap/PLA-SN, PHB/PCL-SN, and PHB/PCL-SN-TCP, respectively. Their mechanical properties (strengths ~40 MPa, moduli ~2.5 GPa) were comparable with human trabecular bone. The calculated surface energies of all blends were ~40 mN/m. Unfortunately, only two out of three materials were proven to be non-cytotoxic (both PHB/PCL blends).

## 1. Introduction

Bioplastics and biodegradable polymers are attracting attention not only for their eco-friendliness but also for their biocompatibility. This quality allows them to be implanted into the human body without causing harm and later absorbed and fully disintegrated. As a result, these polymers are being used in medicine and medical applications to improve regeneration, restore damaged tissues, and shorten healing time. Among the polymers utilized in medical applications are poly(3-hydroxybutyrate), polylactic acid, poly(glycolic) acid, and polycaprolactone, as well as their blends and copolymers [1,2].

Tissue engineering is a highly advanced method utilized in modern regenerative medicine. The primary component of tissue engineering is a scaffold, which is a porous structure that provides support for various cells and becomes integrated into the human body. Among the most prevalent health problems, bone damage remains a significant concern. Osteoporosis and traumatic fractures, particularly in older individuals, are common causes of bone damage [3,4]. The scaffold material used in bone tissue engineering must possess certain properties that are essential for successful bone growth at the implant site. Firstly, the material must be osteoconductive, which means it should facilitate bone growth. Additionally, it must be osteoinductive, meaning it should induce the differentiation of local stem cells that are present in the bone marrow into osteogenic cells. For the cells to form new tissue, the scaffold material should have appropriate porosity and a 3D structure that allows cells to grow through the entire volume of the scaffold [5,6,7]. High demands are placed on scaffold properties, making 3D printing the ideal method to meet these requirements. The use of 3D-printed scaffolds has shown great potential in improving treatment for certain medical conditions, shortening healing time, and reducing waste production. Tissue engineering has recently incorporated 3D printing to produce scaffolds from biocompatible polymer blends, allowing for enhanced properties. Among the most commonly used polymers for 3D printed scaffolds are biodegradable ones, including polylactic acid, poly(3-hydroxybutyrate), and polycaprolactone [8,9].

Poly(3-hydroxybutyrate) (PHB) is a biopolymer that is synthesized by bacteria and is highly crystalline. It is also biodegradable under natural conditions [10]. PHB has properties that are comparable to those of polypropylene (PP), including similar melting temperature, glass transition temperature, crystallinity, and tensile strength. However, PHB is considerably more brittle than PP [11,12].

PHB is the most published and commercially successful polyhydroxyalkanoate polymer, which was found to be miscible with other biodegradable polymers such as polycaprolactone (PCL) or polylactic acid (PLA) at different ratios and with inorganic particle fillers such as montmorillonite nanoclay, kaolin, or tricalcium phosphate (TCP) [13,14,15,16,17]. Blending PHB with another biopolymer or the addition of filler is a very effective approach to improve the printability of prepared materials. TCP, as well as hydroxyapatite (HAp), have high bioactivity and have been used as bone substitute materials. Furthermore, mesenchymal stem cells (MSCs) can be induced to differentiate into osteogenic lineages in vivo using tricalcium phosphate (TCP) [18,19,20,21].

Polycaprolactone (PCL) is widely used in tissue engineering to manufacture different types of stents due to its good biocompatibility and shape retention properties. PCL has a low melting point, making it ideal for 3D printing, and it has been extensively studied as a scaffold material for tissue engineering due to its ability to support cell attachment, proliferation, and differentiation [22,23,24]. PCL scaffolds have been used for a variety of tissue engineering applications, including bone regeneration [25,26,27,28]. PCL scaffolds have been shown to have several advantages over other scaffold materials [29]. PCL degrades slowly over time, which allows for the gradual release of growth factors and other signaling molecules that can promote tissue regeneration. Additionally, PCL has been shown to have good mechanical properties, which can help maintain the structural integrity of the scaffold over time [30,31].

Tissue engineering and regenerative medicine face several challenges, particularly in bone tissue engineering, which hinder their progression to clinical practice. The main challenges are the need for a synergistic combination of biomaterials, cells, and factor therapy to induce new functional bone regeneration, the development of widely investigated biomaterial scaffolds with appropriate micro- and nano-structural properties, and the incorporation of biomimetic properties and growth factors. Other challenges include improving the biofunctionality, antimicrobial behavior, biocompatibility, and stem cell differentiation efficiencies of implants. Bone biofabrication using additive manufacturing techniques is suggested as a possible solution to these challenges. Additionally, animal and human trials are essential, and there is a need for close collaboration between medical doctors and biomaterials scientists to address critical problems and advance the field [32,33,34,35].

This work focuses on research on the 3D printing of scaffolds based on PHB biodegradable polymeric blends for tissue engineering and bone medical applications. The objective is to create a material that possesses appropriate thermal and mechanical properties, making it capable of being processed through FDM 3D printing. Additionally, it is essential for the material to be non-cytotoxic while also having the potential to be osteoconductive, osteoinductive, or osteogenic. The influence of material composition on cell proliferation was also established [36].

## 2. Materials and Methods

### 2.1. Materials

A total of three composite polymer blends were used in the research. Two blends were based on a plasticized blend of poly(3-hydroxybutyrate) (PHB) EDW 2394 from TianAn (Ningbo City, China) and polycaprolactone (PCL) Capa^®^ 6500 from Perstorp (Malmö, Sweden) with the same composition as the PHB/PLA blends studied in our previous work [37]. The third material was based on poly(3-hydroxybutyrate) produced by the chloroform-free soap route (abbreviated as PHB-soap) [38] and amorphous poly(d,l-lactide) (PLA) Ingeo 4060D from NatureWorks (Minnetonka, MN, USA). All three materials were plasticized using Syncroflex TM 3114 (oligomeric adipate ester, abbreviated as SN) from Croda (Rawcliff, UK), and a tricalcium phosphate blend (αTCP = 7%, βTCP = 93%, abbreviated as TCP) from CN Lab Nutrition (Shaanxi, China) was added to the matrix as a bioactive filler. The mean particle size of TCP was 10.8 μm measured by laser diffraction using a HELOS analyzer by Sympatec GmbH (Remlingen, Germany). The composition of prepared biocomposites is given in Table 1.

The extrusion process was performed using a corotating meshing twin screw extruder from Labtech Engineering Company (Samutprakarn, Thailand) (D = 16 mm, and L/D = 40), with the temperature setting of individual zones 80–180–180–180–180–175–170–160–160–160 °C from a hopper to a nozzle and a rotational speed of the screw set to 150 rev.·min^−1^. The total weight of each sample for extrusion was 500 g.

### 2.2. 3D Printing Filaments

The HAAKE™ Rheomex OS single screw extruder (Haake Technik GmbH, Vreden, Germany) was used to process granules of prepared materials into 3D printing filaments. The chamber temperature profile from a hopper to a nozzle was set at 185–175–170–160 °C, while the screw’s rotational speed was maintained at 20 rev.·min^−1^. To ensure a constant diameter of the filament of 1.75 mm, the filament was directed to the calibration unit after being pulled through the water tank tempered to 60 °C, and the draw-off device passed through the air dryer before being wound onto a coil.

### 2.3. Differential Scanning Calorimetry (DSC)

Three newly prepared polymer blends, namely PHB-soap/PLA-SN, PHB/PCL-SN, and PHB/PCL-SN-TCP, were subjected to differential scanning calorimetry (DSC) to evaluate their thermal properties. The samples were measured in two forms, granulate (marked as “_G”) and prepared 3D printing filament (marked as “_F”).

DSC measurements were conducted on DSC 2500 (TA Instruments, New Castle, DE, USA) under a nitrogen atmosphere. The samples were first cooled to −30 °C and then subjected to two heating cycles to 200 °C with a heating rate of 10 °C·min^−1^. Aluminum pans with samples were hermetically sealed before the measurement, and the polymer sample masses were approximately 10 mg.

The crystallinity of the samples was calculated using Equation (1), where ∆*H*_m_ and ∆*H*^0^_m_ (J/g) are the enthalpy of fusion from the second heating cycle and the enthalpy of fusion of 100% crystalline polymer (146 J/g for PHB and 139 J/g for PCL), respectively:

(1) $$X_{c}=\frac{\Delta H_{m}}{\Delta H_{m}^{0}}\cdot 100\;\left(\%\right)$$

### 2.4. Parameters of 3D Printers

The specimens were printed using Original Prusa i3 MK3S and MK3 FDM 3D printers controlled by V3.9.0 firmware version, manufactured by Prusa Research a.s. in Prague, Czech Republic. The MK3S printer, equipped with a 0.25 mm diameter nozzle, was utilized for printing specimens intended for optical contact angle measurement (OCA), surface roughness analysis, and in vitro tests on scaffolds. On the other hand, the MK3 printer, which features a 0.4 mm diameter nozzle, was used for printing specimens intended for the temperature tower test, warping test, tensile test, three-point flexural test, and compression test.

All G-codes were prepared in PrusaSlicer, software version 1.41.2. PrusaSlicer is based on an open-source platform called Slic3r, licensed under the GNU Affero General Public License, version 3. The print profile used was taken from the profile for PLA. Therefore, the printed sample was cooled by a fan at 100% from the second layer. The flow value of 100% was used for all samples, except when the prepared 3D printing filaments tended to be a little bit thicker than the desired 1.75 mm, in which case the flow was changed to 95%.

The maximum printing space of both printers is 250 × 210 × 200 mm (x × y × z), with the resolution of the x and y-axes being 10 µm and the resolution of the z-axis being 5 µm. The basic printing parameters of the MK3S and MK3 printers are provided in Table 2.

### 2.5. Optimization of 3D Printing Temperature

The 3D printing filaments underwent tests to assess their printability and determine the ideal processing conditions. The temperature tower test (TTT) was used to optimize the temperature of the nozzle. The test involved three towers, with the first tower having a temperature range of 220–195 °C, the second tower having a range of 195–170 °C, and the third tower having a range of 175–150 °C. The temperature range started with the highest temperature at the bottom and gradually decreased by 5 °C to the lowest temperature at the top. A separate article provides further details [39].

### 2.6. Determination of Warping Coefficient

There were two aspects to optimizing warping during 3D printing. First, the optimal printing temperature (or temperatures) was determined based on the TTT, along with one or two additional temperatures 5 °C above and below. To test for warping, a smaller value of the warping coefficient was preferred. Further information about this test is provided in our previous publication [39].

Once the optimal printing temperature for a particular material was determined, it was used for all subsequent printings of that material. The first temperature of the pair was used to print the brim and the first layer of the specimen, while the second temperature was used for printing all remaining layers.

For PHB-soap/PLA-SN, the flow was set to 100%, the fan was disabled, and the printing speed was set to 80%. This ensured that each printed layer had enough time to cool down before the next layer was printed.

### 2.7. Compression Moulding and 3D Printing of Testing Specimens

The process of compression molding was employed to produce granules of composite materials using a hydraulic laboratory press Qnubu Rosin Press (Qnubu, Parets del Vallès, Spain). To begin the process, 5 g of granulate was evenly distributed on the press plate. A spacer grid with a thickness of 1 mm was used to determine the final thickness of the plate. The press plates were inserted between the top and bottom platforms that were heated to a temperature of 190 °C. The molding process took 100 s under a pressure of 20 MPa. Once the process was complete, the press plates were removed, and the molded plate was allowed to cool at laboratory temperature for one minute. Finally, the press plates were rapidly cooled under running water until the molded plate could be removed.

### 2.8. 3D Printing of Testing Specimens

The original PrusaSlicer software (Prusa Research a.s., Prague, Czech Republic) was used to create the G-codes for 3D printing all specimens. Standardized double-paddle testing specimens (dogbones 5A) with a cross-section of 4 × 2 mm, according to the CSN EN ISO 527-2, were printed for tensile testing. The G-code for 3D printing the dogbones involved regular alternating of two layers. The first layer was created by importing the fabricated model to the PrusaSlicer software and adjusting the perimeters to form the entire neck of the dogbone. The second layer was formed manually by inserting infill parallel to the longest dimension of the dogbone. A separate article described the development of this G-code [40].

For the three-point flexural test, rectangular testing specimens with dimensions of 80 × 10 × 4 mm were printed. The G-code was created so that the entire volume of the specimen consisted exclusively of perimeters. In cases where specimens contained PCL, the bed was heated to 60 °C to facilitate removal.

Rectangular testing specimens with dimensions of 10 × 4 × 10 mm were printed for the compression test. Testing specimens for all the mechanical tests were printed using all five polymer blends prepared. In cases where specimens contained PCL, the bed was heated to 60 °C to facilitate removal after the end of printing.

### 2.9. Mechanical Testing

To evaluate the properties of the prepared materials, three mechanical tests were conducted, including tensile, three-point flexural, and compression tests. The measurements were carried out 7 days after the sample preparation to ensure the completion of the material crystallization process. The average of at least six measurements was taken for each test, and the results were presented as mean ± SD.

For the mechanical tests, the ZwickRoell Z010 device from ZwickRoell GmbH & Co. (Ulm, Germany). was used. The tensile, flexural, and compression tests were conducted according to the CSN EN ISO 527, CSN EN ISO 178, and CSN EN ISO 604 standard methods, respectively. The details of the testing conditions for tensile and flexural tests can be found in our previous article [37].

In the compression test, a load indicator with a maximum force of 10 kN was utilized, and the preload was set to 1 N. The compression modulus was determined from the deformation range of 1.25–1.50%. The test speed was set to 1 mm·min^−1^, and the limit deformation for the end of the measurement was set to 10%.

### 2.10. Optical Contact Angle Measurement (OCA) and Surface Energy Evaluation

The surface energy of all five materials was determined by measuring molded plates made from their melt. Contact angles and their standard deviations were used to calculate surface energy, including dispersive and polar components, using an OCA 10 (Dataphysics, Filderstadt, Germany) with SCA-2000 software (Xi’an, China). Four liquids were used in the measurement, including water, diiodomethane, formamide, and diethylene glycol, with water and diethylene glycol acting as polar liquids and diiodomethane and formamide as nonpolar liquids. The average of at least ten drops was taken to determine the results, which are presented as mean ± SD. The contact angle with a certain liquid indicates the degree of effort required by the liquid to minimize its contact with the surface.

The OWRK method (Owens, Wendt, Rabel, and Kaelble) was employed, and results for each liquid were added according to the corresponding research methodology, including Rabel for water, Owens et al. for diiodomethane, Rabel for formamide, and Erbyl for diethylene glycol.

### 2.11. Cytotoxicity Measurement

To determine whether the newly prepared materials (PHB-soap/PLA-SN, PHB/PCL-SN, and PHB/PCL-SN-TCP) were suitable for use in tissue engineering, cytotoxicity tests were conducted on these materials. The materials were sterilized with ethylene oxide, and the culture of their extracts was used with osteoblastic cell line MC3T3-E1 fibroblasts to evaluate their biocompatibility. The cells were subjected to metabolic activity assay and visualization using optical microscopy. The biocompatibility of the samples is essential for their use in tissue engineering applications. The test was performed in accordance with EN ISO 10993.

The extracts were prepared by incubating the samples (6 mm in diameter, sterilized using ethylene oxide) in growth medium DMEM (Sigma Aldrich) supplemented with 10% fetal bovine serum and 1% antibiotics (Penicillin/Streptomycin) at 37 °C for 48 h, 250 µL/sample. 3T3 mice fibroblasts were seeded in a growth medium in a 96-well plate with a density of 2 × 10^3^ cells per well and cultured with the extracts for 24 and 72 h at 37 °C and 10% CO_2_. After 24 h, 200 µL of the extract was added to each well.

Cell morphology and proliferation were observed using optical microscopy 24 and 72 h after seeding (microscope Olympus IX51, camera Olympus DP80), and the MTS assay (CellTiter 96^®^ AQueous One Solution Cell Proliferation Assay; Promega) was performed to assess cell viability. Cells were incubated in MTS solution (20 µL) and 100 μL of the fresh culture medium at 37 °C for 2 h. The MTS substrate was metabolized by mitochondrial enzymes to violet formazan, which absorbs light at 490 nm. The absorbance of the solution (100 µL) was measured using a microplate reader (Infinite M200 PRO, Tecan, Switzerland) at 490 nm and reference wavelength 690 nm. The absorbance of the pure medium with MTS substrate was subtracted from the values.

The MTS test uses a new type of tetrazolium dye, which ultimately results in an aqueous, soluble formazan product. The tetrazolium dye used in the MTS assay is 3-(4,5-dimethylthiazol-2-yl)-5-(3-carboxymethoxyphenyl)-2-(4-sulfophenyl)-2H-tetrazolium. The formation of the soluble formazan dye occurs in the presence of phenazine methosulfate (PMS), which serves as an intermediate electron acceptor, transferring electrons from NADH to reduce the tetrazolium dye, forming the soluble formazan product. The main difference between MTT and MTS assay is that MTT assay has an additional step associated with the solubilization of formazan crystals, whereas MTS assay is not associated with the solubilization of formazan crystals. Therefore, the MTS test is more effective and less time-consuming.

The MTS assay was performed on six independently prepared samples, and the data were statistically evaluated using SigmaStat 12.0 (Systat, Palo Alto, CA, USA). Quantitative data are presented as mean values ± standard deviation (SD). The statistical significance between a pair of groups was determined by the ANOVA test and Student–Newman–Keuls test for post hoc analysis if the data passed the normality test and the test of equality of variances. If the data were without normal distribution, statistical significance between a pair of groups was determined using Kruskal–Wallis One Way Analysis of Variance on Ranks and Dunn’s multiple comparisons test for post hoc analysis. A *p*-value less than 0.05 was considered statistically significant. Margin values were not included due to the variability of the measured results, but at least four samples were used for the statistical evaluation.

## 3. Results

### 3.1. Thermal Properties of Prepared Materials and Filaments for 3D Printing

#### Differential Scanning Calorimetry (DSC)

Table 3 displays the crystallization temperatures (*T*_c_) and crystallinities (*X*_c_) of PHB and PCL. The measured melting temperature (*T*_m_) of PHB-soap was between 168.2–169.7 °C, while PCL was between 52.0–54.2 °C. Therefore, the melting temperatures were not included in the table. The crystallinity of PHB-soap remained relatively stable in both granulate and filament forms, with only a 0.9% change. Moreover, the thermal properties of PHB-soap, such as the melting and crystallization temperatures, were not affected by the process of preparing 3D printing filament from granulate by extrusion.

When comparing the thermal properties of PHB-soap and commercial PHB, it was found that only the *T*_c_ of the PHB/PCL-SN_G blend was comparable to that of PHB-soap/PLA-SN in granulate and filament form. In all other blends, the *T*_c_ was approximately 10 °C greater than that of PHB-soap. Additionally, the crystallinity (*X*_c_) of commercial PHB blends was always higher than that of PHB-soap.

It was observed that TCP and the process of filament extrusion have a nucleating effect on commercial poly(3-hydroxybutyrate), leading to an increase in *T*_c_. In contrast, TCP does not nucleate PCL, but the process of filament extrusion does, resulting in an increase in *T*_c_ and crystallinity.

### 3.2. Optimisation of Processing Conditions and Printability Testing of Prepared 3D Printing Filaments

#### 3.2.1. Temperature Tower Test (TTT)

**Figure 1:**
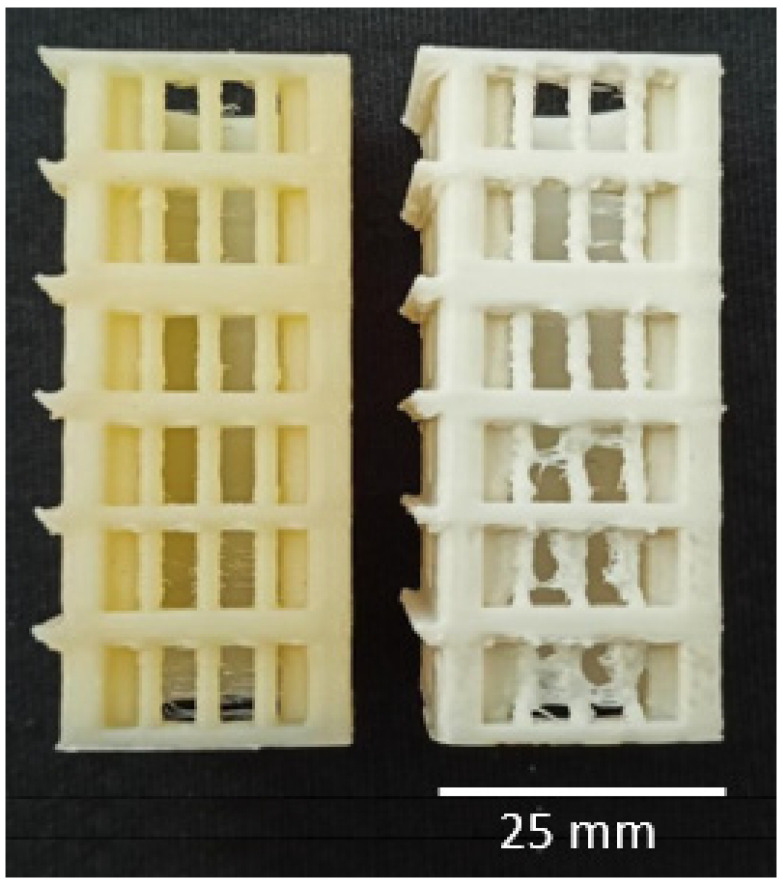
An image of 3D printed temperature towers of PHB-soap/PLA-SN (temperature range 195–170 °C) with optimal quality of printed elements (**left**) and PHB/PCL-SN-TCP (temperature range 220–195 °C) with non-optimal quality of printed elements (**right**).

Table 4 presents all the results obtained from the temperature tower test (TTT). Figure 1 illustrates an example of 3D-printed temperature towers. Each tower provided a range of applicable temperatures, and the quality of the printed geometric elements on each tower was considered to determine the best temperature or range of temperatures for 3D printing. After conducting the TTT, the optimal temperature or temperatures for 3D printing were determined to be 190–185 °C for PHB-soap/PLA-SN, 170 °C for PHB/PCL-SN, and 170–165 °C for PHB/PCL-SN-TCP. These temperatures were then utilized for the warping test.

#### 3.2.2. Warping Test

Table 5 presents the results of the warping test. A linear trend can be observed in the warping coefficient for PHB/PCL-SN, where the coefficient decreases with increasing temperature. However, in the case of the PCL blend with TCP, the warping coefficient increases. It should be noted that the dependency plot was based on only three points, and additional data would be required to further support this observation. Taking into account all the results from the TTT and warping test, the final processing temperatures for the 3D printing of the three prepared composite materials were determined. The processing temperatures selected were 195/190 °C for PHB-soap/PLA-SN, 195/175 °C for PHB/PCL-SN, and 195/165 °C for PHB/PCL-SN-TCP.

### 3.3. Mechanical Properties

#### 3.3.1. Tensile Test

**Figure 2:**
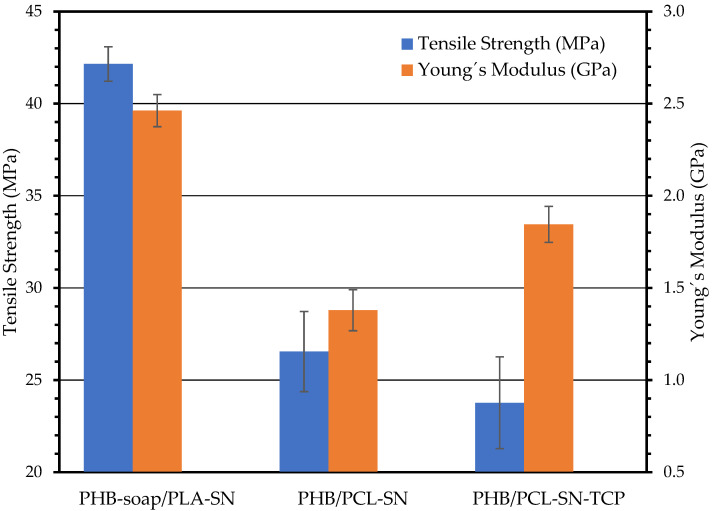
The results of tensile test of 3D printed dogbones.

The results of the tensile test are presented in Figure 2, which illustrates that the addition of TCP to the PHB/PCL blend results in a reduction of the blend’s tensile strength by 10.5%. Additionally, the ductility of the blend decreases as the TCP performs the function of a stress concentrator. On the other hand, the addition of TCP increases Young’s modulus by 28.6%, which is typical for fillers.

In comparison to their PHB/PLA counterpart, PHB/PCL blends exhibit lower values of measured characteristics because PCL is more ductile at room temperature than PLA due to its lower glass transition temperature (*T*_g_) of −60 °C compared to PLAs 60 °C, which results in more ductile specimens.

#### 3.3.2. Three-Point Flexural Test

**Figure 3:**
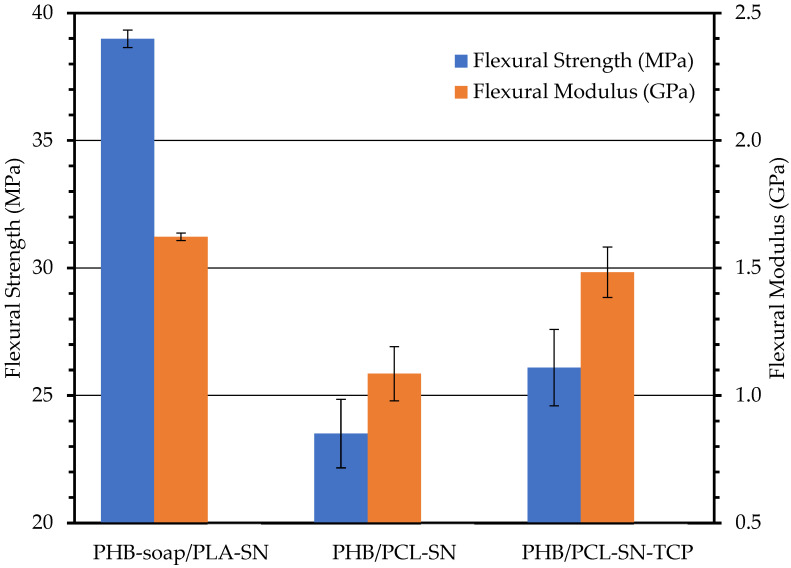
The results of a three-point flexural test of 3D printed specimens.

The graph in Figure 3 presents all the results obtained from the flexural test. As in the case of the tensile test, PHB/PCL blends do not reach such mechanical properties in comparison to PHB-soap/PLA. The addition of TCP to the PHB/PCL blend enhanced flexural strength by 11.1% and flexural modulus by 36.4%, providing reinforcement against bending stress. These results confirm the assumption that PCL blends exhibit lower mechanical properties than PLA blends due to the lower glass transition temperature of PCL and that the addition of a bioactive filler (TCP) helps to at least partially improve these mechanical properties.

#### 3.3.3. Compression Test

**Figure 4:**
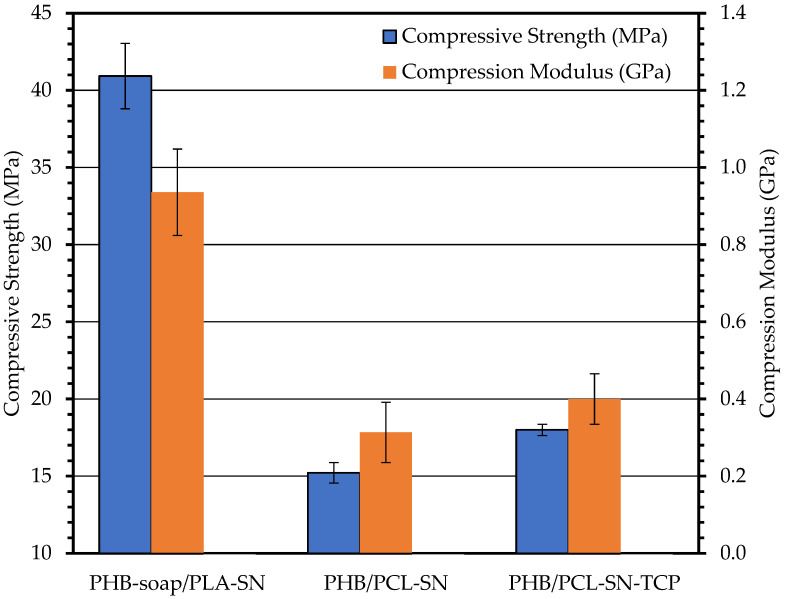
The results of compression test of 3D printed specimens.

The compression test results are illustrated in Figure 4, showing that the addition of TCP to the PHB/PCL blend significantly increases compressive strength and compression modulus. The reinforcement of the specimens against pressure accounts for the improvement in mechanical properties. Compared to PHB/PCL blends, PHB/PLA blend exhibits higher mechanical properties, as confirmed by the three tests.

In terms of mechanical properties, the PHB/PLA blend displays the highest strength during compression testing, although the strengths during tensile and flexural tests are also comparable to those during compression tests. During the tensile test, the specimens show the highest modulus, while the flexural and compression moduli are relatively lower.

When comparing the mechanical test results with human trabecular bone, which is a porous and relatively poorly calcified material, the moduli can range between 0.01–3 GPa, and the strengths between 0.1–30 MPa, depending on the bone type, its location in the body, and the orientation of applied stress [41].

The addition of TCP into the blends has a positive impact on Young’s modulus, flexural strength, flexural modulus, compressive strength, and compression modulus. However, it decreases the tensile strength and ductility of the 3D-printed specimens of the PHB/PCL blend. The positive influence on mechanical properties is more significant and critical for scaffolds intended for bone tissue engineering, even though the ductility decreases.

### 3.4. Surface Properties and Their Influence on Cell Adhesion

#### Optical Contact Angle Measurement (OCA) and Surface Energy Calculation of Prepared Materials

**Figure 5:**
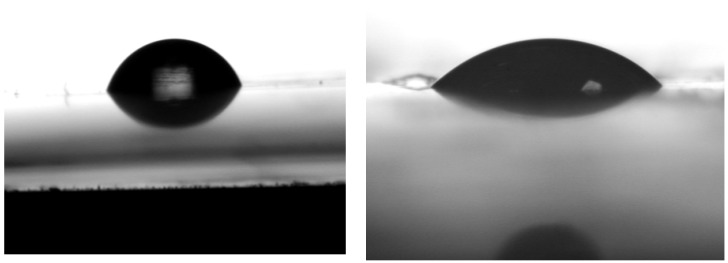
An image of PHB-soap/PLA-SN surface with a water drop (**left**) and PHB/PCL-SN-TCP surface with diethylene glycol (**right**).

Table 6 presents the results of optical contact angle measurement (OCA) conducted on molded plates of all three composite materials. The contact angle of water was the greatest among all liquids used, with a value of 84.6° for the PHB-soap blend. This indicates that all prepared materials exhibit nonpolar hydrophobic character, repelling polar liquids. Diethylene glycol, the second polar liquid used, showed the second-greatest contact angle only in the case of the PHB-soap/PLA blend. However, the contact angles of all other remaining liquids were very similar to that of diethylene glycol, with a difference of only approximately 2°, probably due to its comparatively lower polarity than water. In three of five cases, the lowest contact angle was observed for diiodomethane, with PHB/PCL-SN exhibiting the lowest contact angle of just 22.9°. This further confirms the hydrophobic nature of all five materials, as diiodomethane is the least polar of all four liquids used. Figure 5 illustrates the difference between good and poor wetting.

All materials had a very similar surface energy of around 40 mN/m, with a dispersive component of approximately 30 mN/m and a polar component of 10 mN/m, similar to PMMA or PEO [42]. PHB-soap/PLA-SN showed a dispersive component 5 mN/m greater and a lower polar component by the same amount compared to all other materials, indicating even less polarity and more hydrophobicity. The calculated surface energies of all materials (40 mN/m) were found to be suitable for cell growth and proliferation according to previously published studies [43,44].

The surface energy of PHB homopolymer is 47 mN/m with a dispersive component of 38 mN/m and a polar component of 9 mN/m [45]. On the other hand, the surface energy of PLA is 36 mN/m with a dispersive component of 27 mN/m and a polar component of 9 mN/m [46], while PCL has a surface energy of 31 mN/m with a dispersive component of 26 mN/m and a polar component of 5 mN/m [47].

### 3.5. Cytotoxicity Measurement

**Figure 6:**
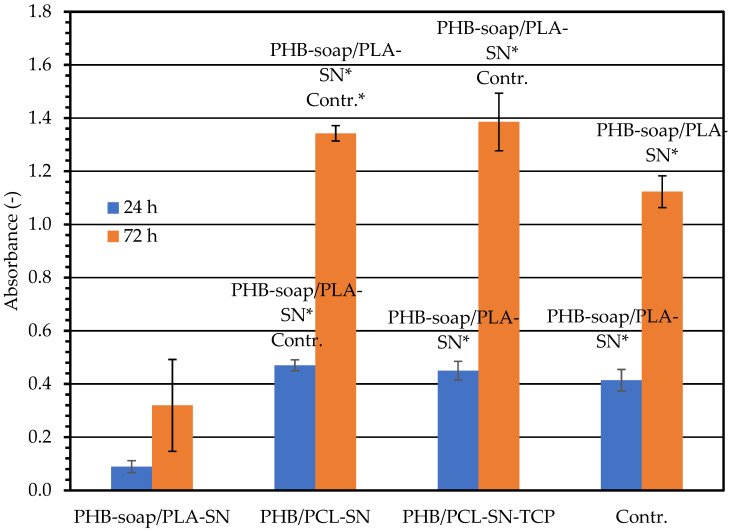
Metabolic activity of 3T3 fibroblasts cultured in extracts measured using MTS test 24 and 72 h after seeding. 3T3 fibroblasts cultured in growth medium were used as a control (Contr.). The labels above the columns sign statistical significance (*p* < 0.05, * *p* < 0.001).

The impact of the extracts of the materials on 3T3 fibroblasts was used to determine their cytotoxicity. After the addition of the extracts, the metabolic activity of the cells was evaluated by performing the MTS test at 24 and 72 h, as presented in Figure 6. PHB-soap/PLA-SN exhibited the lowest absorbance value after 24 h, while the blends of PHB/PCL had nearly equivalent absorbance values. The PHB/PCL-SN-TCP absorbance value was comparable to that of the control group. Meanwhile, the value of PHB/PCL-SN was statistically higher. The blends of PHB/PCL showed increased metabolic activity, with absorbance levels higher than those of the control group. However, PHB-soap/PLA-SN exhibited very low metabolic activity and displayed cytotoxicity.

The cells in the control group that were cultured in a normal medium appeared healthy, and their population increased up to day 3, as presented in Table 7. The cells that were cultured in extracts of PHB/PCL-SN and PHB/PCL-SN-TCP displayed good morphology and formed a confluent layer after 72 h. The group with PHB-soap/PLA-SN extracts exhibited fewer cells compared to the other groups, and even though some of the cells in this group proliferated, the rest underwent apoptosis.

## 4. Discussion

Optimal printing temperatures of 195/190 °C for PHB-soap/PLA-SN, 195/175 °C for PHB/PCL-SN, and 195/165 °C for PHB/PCL-SN-TCP were identified. The measured melting temperature (*T*_m_) of PHB-soap was found to be between 168.2–169.7 °C, while that of PCL was between 52.0–54.2 °C. It was observed that TCP and the process of filament extrusion had a nucleating effect on commercial poly(3-hydroxybutyrate), leading to an increase in *T*_c_. Despite the fact that the mechanical properties of PHB/PCL blends were lower than those of PHB-soap/PLA in all mechanical tests, they still compared favorably with human trabecular bone. These blends exhibited strengths of approximately 40 MPa and moduli of around 2.5 GPa. Furthermore, the materials prepared were found to be hydrophobic, with surface energies of approximately 40 mN/m. Unfortunately, PHB-soap/PLA-SN was found to be cytotoxic, while PHB/PCL-SN and PHB/PCL-SN-TCP were non-cytotoxic and biocompatible. Therefore, these two PHB/PCL blends hold great potential for future research in tissue engineering.

Ecker et al. developed blends of polylactic acid (PLA) with an amorphous copolymer of polyhydroxyalkanoates (PHA), where the specific composition of the PHA copolymer was not disclosed. The temperature used for 3D printing was 205 °C for the entire sample, and the printing bed was heated to 80 °C. The resulting mechanical properties of the PLA/PHA blend, with tensile strengths of approximately 40 MPa and Young’s moduli of 2.5 GPa, were comparable to those observed for the novel material PHB-soap/PLA, which was prepared by a chloroform-free extraction process. Furthermore, the crystallinity of the PLA/PHA blend was found to be approximately 55%, which is consistent with the observed thermal properties of the PHB-soap/PLA blend. These findings suggest that PHB-soap extracted by the chloroform-free process exhibits similar properties to commercially available PHB extracted using traditional solvents. Moreover, the printing temperature of PHB-soap/PLA was only 10 °C lower than the PLA/PHA blend, and bed heating was not required, indicating that PHB-soap/PLA may be a promising candidate for 3D printing applications [7].

Abdelwahab et al. investigated the preparation of a blend consisting of 75 wt% polylactic acid (PLA) and 25 wt% poly(3-hydroxybutyrate) (PHB), which were plasticized with 5 and 7 wt% of a polyester plasticizer, Lapol 108. The resulting blends exhibited crystallinity values ranging from 36 to 40%. However, despite the attempts to improve the mechanical properties of the blends, the tensile strength and Young’s modulus remained poor, measuring only 15 MPa and 1.2 GPa, respectively [48].

In this study, Wang et al. investigated the potential to enhance the mechanical properties of PLA filaments by blending them with PHB in an 80/20 weight ratio. The optimal 3D printing temperature for the PLA/PHB blend was found to be 190 °C, which was lower than that of neat PLA (210 °C). The presence of PHB domains resulted in a decrease in Young’s modulus and tensile strength from 3.6 GPa to 3.4 GPa and from 66 MPa to 55 MPa, respectively. However, annealing the samples resulted in a significant improvement in their mechanical properties, with Young’s modulus even exceeding that of neat PLA [49].

Laoutid et al. prepared PHB/PCL blends by reactive extrusion. In this study, the mechanical properties of PHB/PCL blends were significantly improved by the addition of DCP as a compatibilizing promotor and grafting agent, as well as low molecular weight PEG as a plasticizer. The resulting materials showed that the thermal stability of the blend was slightly improved, with a bimodal PHB melting endotherm indicating the occurrence of grafting and crosslinking reactions. With the ratio of PHB/PCL shifted from 80/20 to 60/40, Young’s modulus decreased from 1.4 GPa to 700 MPa and tensile strength from 34.5 MPa to 19.1 MPa. PCL is above its *T*_g_ at ambient conditions, which results in the deterioration of mechanical properties with higher PCL content [50].

Regrettably, none of the studies presented in this research delved into the biocompatibility of the prepared blends. Their main focus was on enhancing processing techniques and mechanical properties. Nonetheless, the novel materials showcased in this study possess a significant advantage in terms of their properties being similar to, or even better than, those examined in previous publications.

These findings propose that PHB/PCL blends hold great potential for future research in bone regeneration applications, as they can substitute or aid damaged bone tissues. Additionally, leveraging 3D printing technology to develop patient-specific implants and scaffolds using these materials could boost the success rates of bone regeneration surgeries and reduce the need for invasive surgeries and implant rejection. Furthermore, the study’s results emphasize the importance of conducting thorough cytotoxicity testing for new biomaterials to guarantee their biocompatibility and safety for use in medical applications. Taken together, the findings of this study represent a promising step toward the development of novel biomaterials for tissue engineering and medical bone applications, with the potential for future clinical translation.

## 5. Conclusions

The objective of this study was to explore the potential of 3D printable polymer blends for bone medical applications and tissue engineering. The objective was to develop a material that possesses appropriate thermal and mechanical properties, making it capable of being processed through FDM 3D printing. Additionally, it is essential for the material to be non-cytotoxic while also having the potential to be osteoconductive, osteoinductive, or osteogenic.

In conclusion, this work presents the preparation and characterization of PHB-based composite blends for bone medical applications and tissue engineering. The PHB used was obtained by both commercial and chloroform-free routes and blended with PLA or PCL, plasticized by oligomeric adipate ester, and supplemented with TCP particles. The blends were processed into 3D printing filaments and tested for their thermal, mechanical, surface, and cytotoxic properties. The best temperatures for 3D printing were determined for each blend, and their mechanical properties were found to be comparable to human trabecular bone. However, only two out of the three blends were non-cytotoxic, indicating further development is required for some of the materials tested. Overall, the results show the potential of these PHB-based composite blends for use in bone medical applications and tissue engineering.

## Figures and Tables

**Table 1:** The composition of prepared and used materials: the basic mixture was composed of 12% of plasticizer, and the remaining 88% is PHB-soap/PLA or PHB/PCL in a ratio of 70/30. For filled samples, 15 wt% of TCP was added to the mixture.

	Amount (wt%)				
Material	PHB or PHB-Soap	PLA	PCL	SN	TCP
PHB-soap/PLA-SN	62	26	0	12	0
PHB/PCL-SN	62	0	26	12	0
PHB/PCL-SN-TCP	54	0	23	10	13

**Table 2:** Basic printing parameters of Prusa i3 MK3S and MK3 FDM 3D printers used for printing all specimens.

Parameter	MK3S	MK3
Filament diameter	1.75 mm	1.75 mm
Nozzle diameter	0.25 mm	0.4 mm
Layer height	0.15 mm	0.2 mm
Width of a printed layer	0.3 mm	0.45 mm
Perimeter printing speed	45 mm·s^−1^	45 mm·s^−1^
Fill print speed	200 mm·s^−1^	200 mm·s^−1^
Bed temperature	20 °C	20 °C
Cooling fan power	100%	100%

**Table 3:** The results of DSC measurement; N/A used where not applicable.

Material	*T*_c_, PHB (°C)	*X*_c_, PHB (%)	*T*_c_, PCL (°C)	*X*_c_, PCL (%)
PHB-soap/PLA-SN_G	78.3	58.9	N/A	N/A
PHB-soap/PLA-SN_F	78.8	58.0	N/A	N/A
PHB/PCL-SN_G	78.4	69.9	2.2	45.7
PHB/PCL-SN_F	87.6	61.5	28.4	59.2
PHB/PCL-SN-TCP_G	88.8	83.5	−2.5	13.5
PHB/PCL-SN-TCP_F	86.5	64.2	25.0	51.3

**Table 4:** The results of the temperature tower test (TTT); floor/floors with the best printed geometric element within a single tower; N/A used when no temperature or interval could be selected as the best.

Material	Temperature Range (°C)	Stringing	Colonnade	Diagonal	Holes in Structure	Last Printed Floor (°C)	Best Temperature (°C)
PHB-soap/PLA-SN	220–195	N/A	N/A	N/A	N/A	195	N/A
	195–170	190–170	190–170	185	185–170	170	190–170
	175–150	175–165	175–170	170	175–165	160	175–170
PHB/PCL-SN	220–195	N/A	N/A	N/A	N/A	195	N/A
	195–170	N/A	N/A	N/A	N/A	170	N/A
	175–150	170	170	170	170	165	170
PHB/PCL-SN-TCP	220–195	N/A	N/A	N/A	N/A	195	N/A
	195–170	N/A	N/A	N/A	195–170	170	170
	175–150	N/A	N/A	165	175–165	165	165

**Table 5:** The results of the warping test (the lowest warping coefficients are highlighted in green).

Material	Temperature (°C)	Average Warping Coefficient ± SD (-)
PHB-soap/PLA-SN	190	2.59 ± 0.08
	185	3.06 ± 0.11
PHB/PCL-SN	175	1.26 ± 0.02
	170	1.30 ± 0.02
	165	1.55 ± 0.03
PHB/PCL-SN-TCP	175	1.81 ± 0.04
	170	1.65 ± 0.03
	165	1.53 ± 0.03

**Table 6:** Optical contact angle measurement (OCA) and surface energy of molded specimens.

Material	Liquid	Contact Angle (°) ± SD	Surface Energy (mN/m)	Dispersive Component	Polar Component
PHB-soap/PLA-SN	Water	84.6 ± 2.6	40	35	5
	Diiodomethane	39.2 ± 1.7			
	Formamide	38.7 ± 2.5			
	Diethylene glycol	42.3 ± 2.0			
PHB/PCL-SN	Water	72.7 ± 3.5	40	30	10
	Diiodomethane	22.9 ± 1.4			
	Formamide	55.5 ± 1.8			
	Diethylene glycol	42.2 ± 1.6			
PHB/PCL-SN-TCP	Water	72.1 ± 4.2	41	30	11
	Diiodomethane	35.1 ± 2.2			
	Formamide	47.7 ± 2.7			
	Diethylene glycol	43.3 ± 0.9			

**Table 7:** The cells cultured in extracts were visualized using optical microscopy 24 and 72 h after seeding; magnification 100×.

Time	PHB-Soap/PLA-SN	PHB/PCL-SN	PHB/PCL-SN-TCP	Contr.
24 h	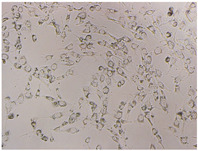	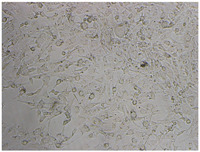	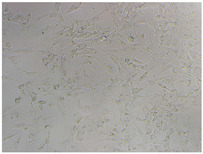	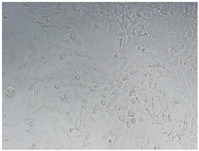
72 h	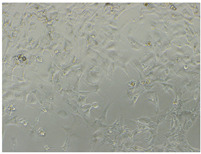	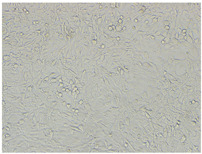	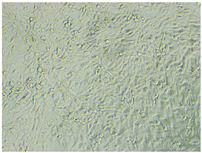	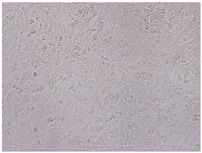

## Data Availability

The data are available upon reasonable request from the corresponding author.
